# Über Cyber

**DOI:** 10.1057/s42984-022-00046-5

**Published:** 2022-10-14

**Authors:** Christian Sievers

**Affiliations:** Academy of Media Arts Cologne, Cologne, Germany

**Keywords:** Cyber war, Information security, Art, Performance, Public space, Participation

## Abstract

A performative art installation in public space. Consisting only of barrier tape and the recording of the German military's “cyber march”, it invites the audience and passers-by to engage with it and fall into the trap of underestimating the amount to which it exerts force on participants.

## Article

In 2017 the German military, the Bundeswehr, opened a new branch of service: “Cyber”, the fourth after Army, Air force, and Navy. To celebrate their new sphere of action, the Bundeswehr had an official march composed. The music has a jolly, rousing quality to it,^1^ sounding like the backdrop to a funfair or carnival, in sharp contrast to the apocalyptic scenarios that have been forecast for an actual all-out war in the information sphere. The march provides the musical backdrop for the artwork, emitted from a small speaker situated in its centre (See video: https://vimeo.com/233447184).

**Fig. 1:**
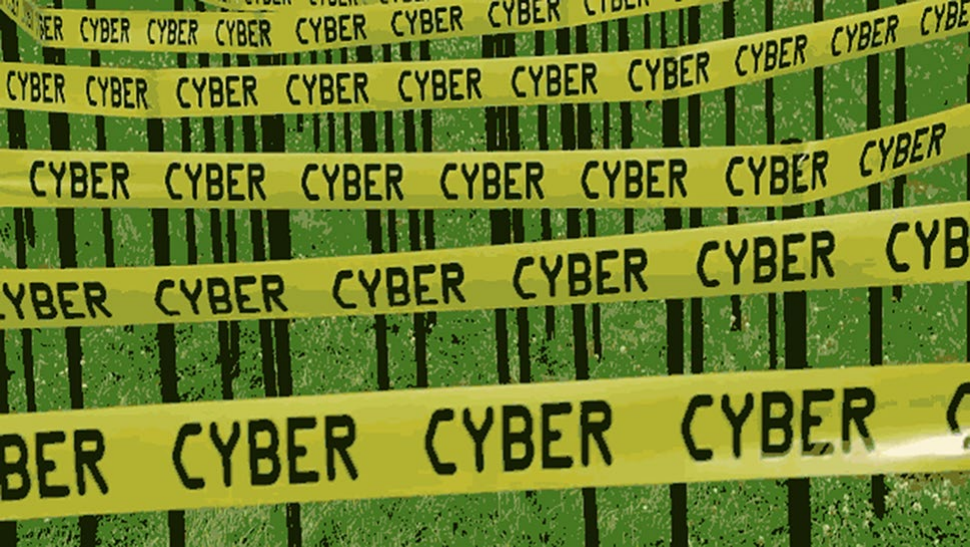
A close-up of the barrier tape

The art installation consists of a spiral^2^ made with barrier tape obtained from a local hacker club. The tape is popular^3^ in Germany’s hacker community which uses the tape as a kind of practical joke device, roping off server cabinets for photograph ops and declaring them safe from hacks—an ironic reference to the fact that in information security nothing can be considered truly unbreachable or inaccessible. In so doing, they are making fun of government information security policies which are regarded as similarly symbolic and ineffective. In German hacker culture, the term “cyber” is understood to be an indicator^4^: those in the know would never in all seriousness employ it. Its usage is associated with political or commercial intent: to stir up fear or in the service of campaigns for stricter laws and wider powers for law enforcement (Fig. 1).

**Fig. 2:**
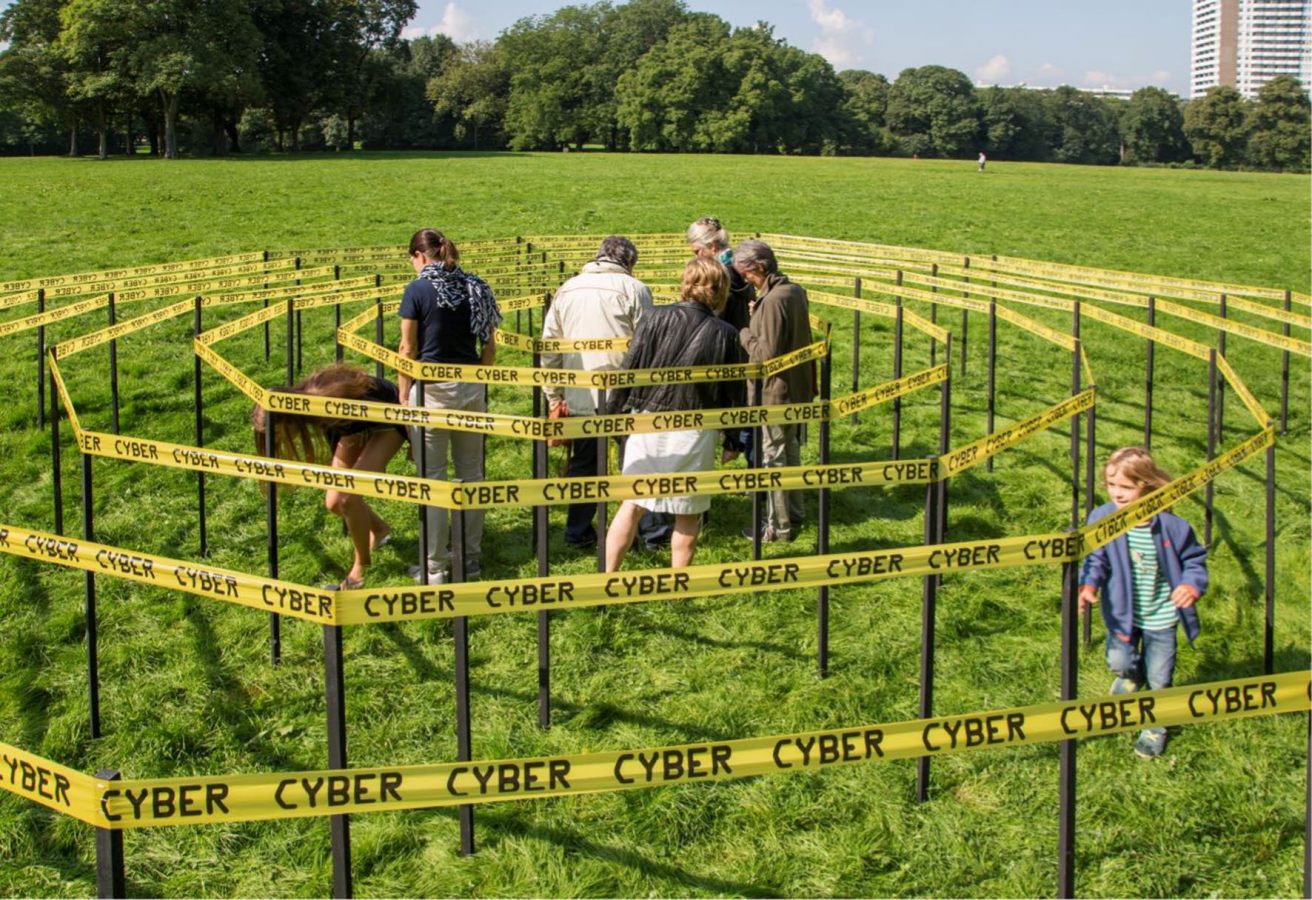
People near the centre of the spiral

As you are approaching the spiral, you are faced with the decision whether to enter it or not. From the outside, it is easy to underestimate its length. The distance from its edge to the centre is only five meters, but the route in is around 180 m long. Walking into it, you slowly realize there is quite a bit of distance to cover, and you soon fall into some kind of automatic trot. March music lends itself to that kind of situation, but the curvy, ever-tightening path requires constant attention. There are people coming towards you, and the corridor is too narrow to easily fit two, so there are traffic jams and blockages. You have to negotiate, make eye contact and make room for each other. It is fun, but awkward. Marching does not work at all (Fig. 2).

The art work is one in a series of participatory installations that function as a kind of trap. In exercising a small degree of coercion, the work reflects the mode in which participation—the central promise of the Internet—takes place on today’s heavily surveilled and weaponized platforms. It is conceivable to refuse, but only at the price of self-exclusion.^5^ Astonishingly, even in their military strategies, democratic Western governments are not free of this dilemma.

**Fig. 3:**
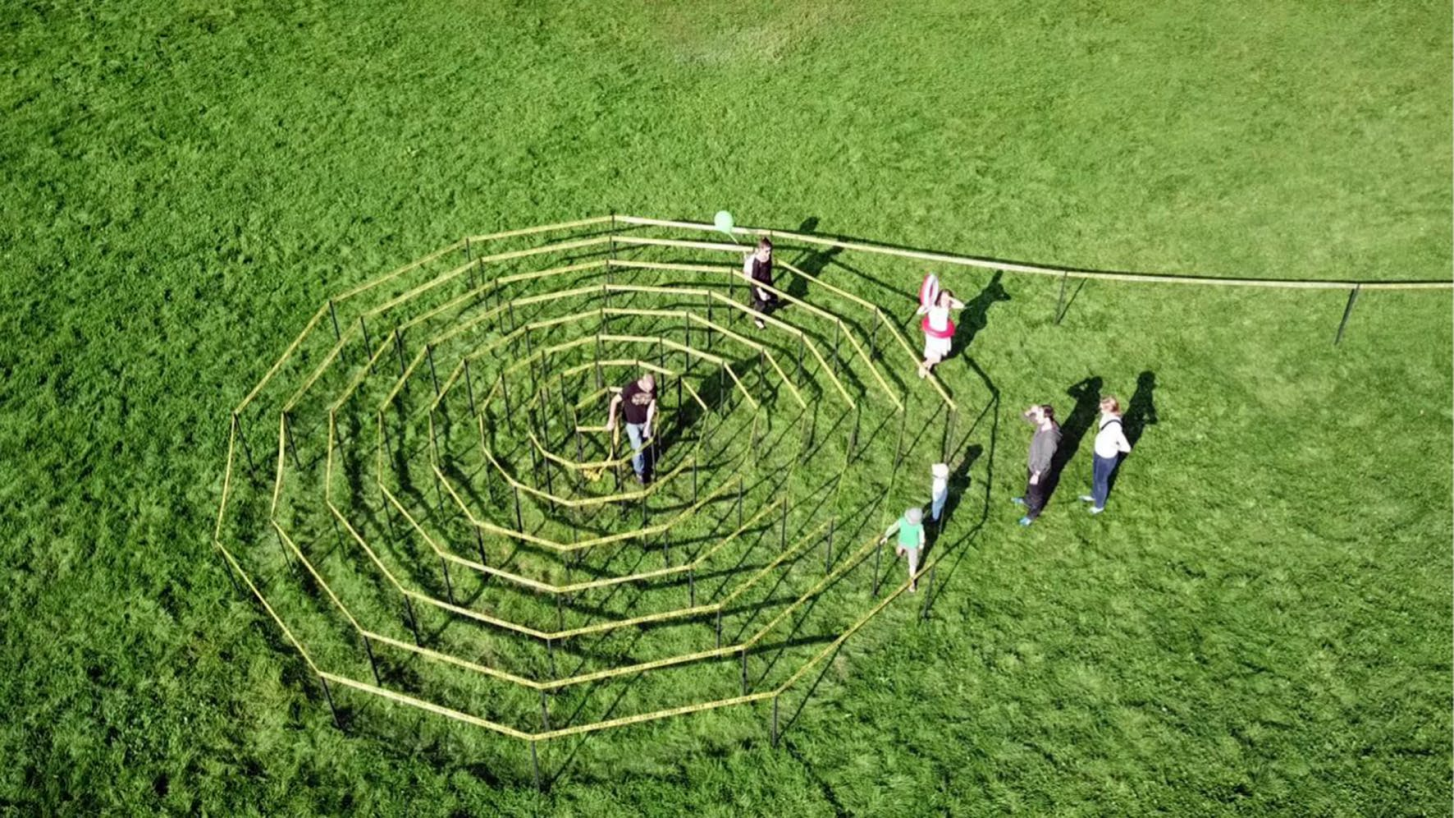
Bird’s eye view

The beginning of my career as an artist coincided with the start of the "war on terror" and the ever-tightening security and surveillance measures that ensued. This process was complimentary and operated in parallel to the increasing application of digital networked technologies to all areas of life. Both developments were accelerated from 2007 by the invention of the smartphone, and again from 2020 by the COVID-19 pandemic. My artistic practice reflects the conditions under which this securitization and digitalization of life is accomplished. The spiral walkway of “Über Cyber” functions as a direct analogy to how this process is experienced (Fig. 3).

## Notes

1. “It is characterized by a powerful rhythm in 6/8 time, a sweeping melody and progressive harmonies. Thus, the character of the march symbolizes the creativity, the spirit of optimism and the visionary drive of the Cyber and Information Space organizational unit." Bundeswehr description of the new march on their public website, where a recording of the march can also be heard https://www.bundeswehr.de/de/organisation/cyber-und-informationsraum/kommando-und-organisation-cir
2. A video documenting the installation can be found here: https://vimeo.com/233447184
3. https://twitter.com/cyberrolle
4. “The use of the word "cyber" is a zero-knowledge-proof.” https://twitter.com/nblr/status/1052680062082924544
5. “This contradiction –voluntarily doing something that one does not really want to do –and the resulting experience of failing to shape one’s own activity in a coherent manner are ideal–typical manifestations of the power of networks.” Stalder, Felix, 2017. *The digital condition*. Cambridge: Polity Press

